# Rare variant of metaplastic carcinoma of the breast: a case report and review of the literature

**DOI:** 10.1186/s13256-017-1553-3

**Published:** 2018-02-21

**Authors:** H. Alaoui M’hamdi, F. Abbad, H. Rais, H. Asmouki, A. Soumani, M. Khouchani, R. Belbaraka

**Affiliations:** 1 Department of Medical Oncology of Marrakech, University Hospital of Marrakech, Marrakech, Morocco; 2 Department of Pathology, University Hospital of Marrakech, Marrakech, Morocco; 3 Department of Gynecology and Obstetrics, University Hospital of Marrakech, Marrakech, Morocco; 4 Department of Radiotherapy of Marrakech, University Hospital of Marrakech, Marrakech, Morocco

**Keywords:** Breast, Metaplastic variant carcinoma, Spindle cell carcinoma, Treatment

## Abstract

**Background:**

Metaplastic carcinoma encompasses a group of neoplasms characterized by differentiation of the neoplastic epithelium into squamous cells and/or mesenchymal-looking elements. Spindle cell carcinoma is a rare variant of this special histological type. Its prognosis remains poor, with a high rate of local recurrence and distant metastasis. To date, only a small number of cases have been described. There is no clear agreement on this histological subtype.

**Case presentation:**

We report a case of a 53-year-old Moroccan woman who consulted our institution following palpation of a nodule of the left breast. Mammography in combination with breast ultrasonography revealed a lesion classified as Breast Imaging Reporting and Data System 4 with microcalcification. The patient was diagnosed with spindle cell carcinoma of the breast. The diagnosis was based primarily on histological and immunohistochemical studies of the breast biopsy and secondarily on the surgical specimen. No local or distant metastasis was found. The treatment used was total surgical excision followed by radiotherapy.

**Conclusions:**

We describe the features (epidemiological, clinical, histological, immunohistochemical, and therapeutic outcomes) of our patient’s case and compare them with literature data.

## Background

Neoplastic lesions of the breast usually arise from atypical proliferation of epithelial cells. Spindle cell carcinoma (SCC) is an exceptional variant of metaplastic carcinoma according to the fourth edition of the World Health Organization (WHO) classification [1]. It is a very rare neoplasm and represents only 0.1% of all mammary malignancies [2]. A few case reports have been published. The diagnosis is based on histology and immunocytochemistry. The origin of SCC has long been a subject of controversy and remains uncertain. However, the epithelial origin is most likely, along with squamous differentiation and myoepithelial participation [3]. The common location of this variant is the parotid gland, but it has been reported in other tissues, such as the salivary gland, vulva, soft tissues, skin, lung, and exceptionally in the breast [4]. The diagnosis, treatment, and outcome are challenging. We report an exceptional clinical case of a 53-year-old woman with SCC of the breast.

## Case presentation

**Fig. 1:**
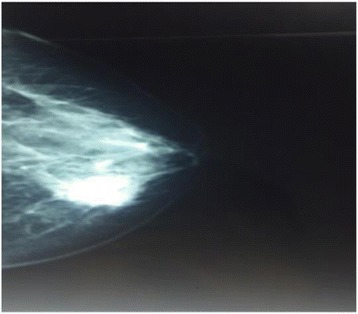
Left mammogram shows lesion with irregular contours and peripheral calcification classified as Breast Imaging Reporting and Data System 3

We report a case of a 53-year-old Moroccan woman with no family history of cancer. She consulted for a lump in her left breast. The initial physical examination revealed a movable and painless nodule measuring 3 cm between the upper and lower inner quadrants in the left breast. There were no inflammatory signs or any retraction of the nipple. The axillary areas were free. Mammography showed a nodular lesion with irregular contours and peripheral calcification. The lesion was classified as Breast Imaging Reporting and Data System (BI-RADS) 4 (Fig. 1). The ultrasonographic examination confirmed the presence of a hypoechoic nodule. The lesion was about 32 mm in size with irregular polylobed contours and located between the superior and internal-inferior quadrants. An ultrasound-guided biopsy was performed. The histopathological examination revealed spindle cell proliferation with no hemorrhage or necrosis. The immunohistochemical (IHC) analysis showed a positive reaction for cytokeratin AE1/AE3 and smooth muscle actin. Ki-67 labeling was 25%, and p63 was positive.

**Fig. 2:**
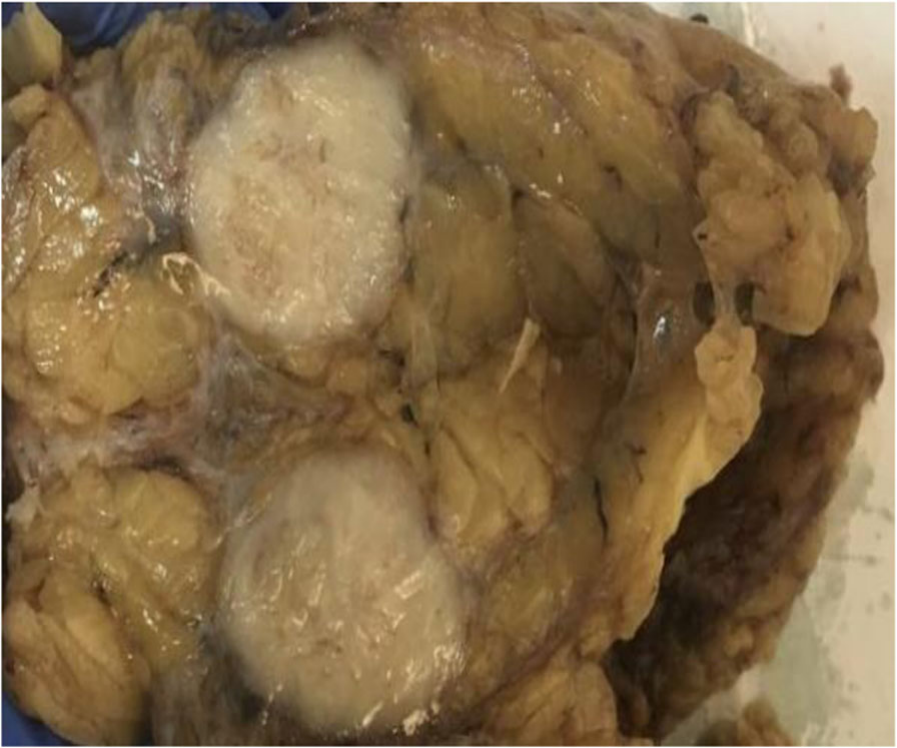
Gross examination of the surgical specimen. A nodular solid tumor is seen between the upper and lower inner quadrants of the left breast

**Fig. 3:**
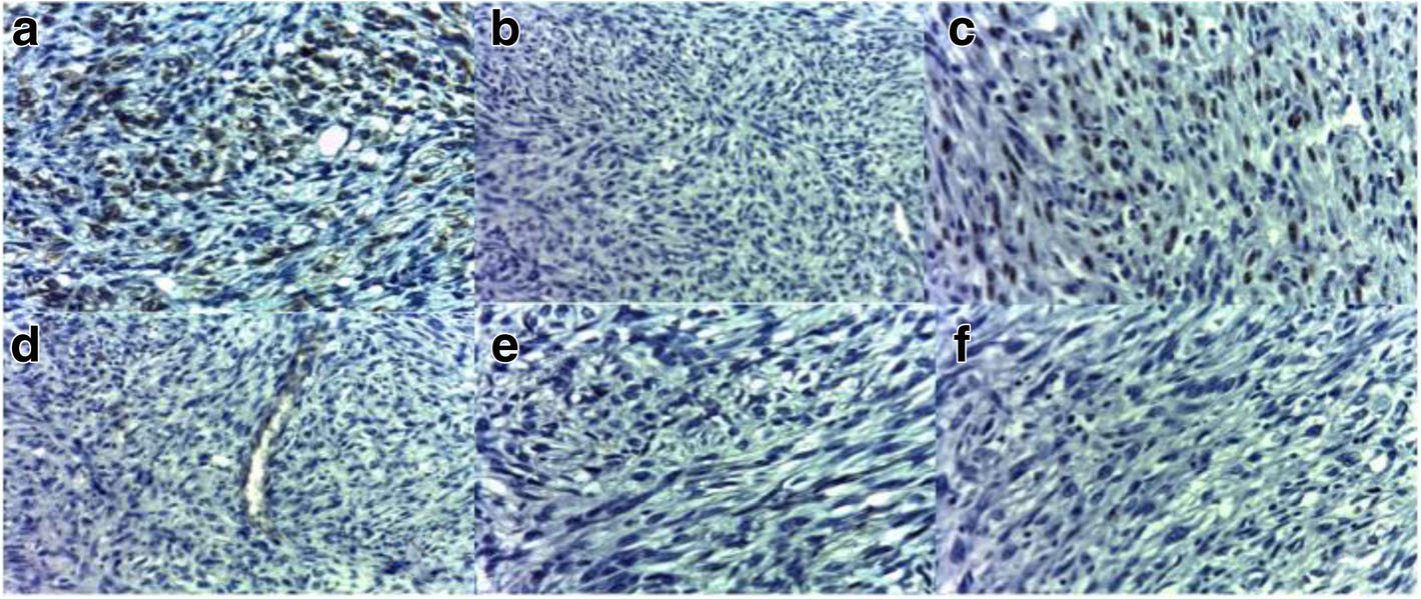
Immunohistochemical findings. **a** Intense membranous expression of cytokeratin C in tumor cells. **b** No expression of cytokeratin 5/6 in tumor cells. **c** Nuclear expression of p63in tumor cells. **d** No expression of CD34 in tumor cells. **e** No expression of CD10 in spindle tumor cells. **f** No membranous staining for human epidermal growth factor receptor 2 antibody

The conclusion of the pathology report was in favor of spindle cell carcinomatous proliferation. After a multidisciplinary assessment, the patient benefited from a radical mastectomy with axillary node dissection. The gross examination of the surgical specimen showed a nodular solid tumor measuring 30 mm between the upper and lower inner quadrants of the left breast (Fig. 2). The closest surgical margin was the posterior one, at 0.2 cm from the neoplasm. A histological examination revealed poorly differentiated spindle cell tumor proliferation. Fifteen lymph nodes were explored, and involvement was found zero node-negative/15 node explored (0 N-/15 N). The IHC examination of the surgical specimen showed focal expression of cytokeratin (AE1/AE3) and moderate cytoplasmic expression of cytokeratin 14 (CK14). The spindle cells also displayed nuclear expression of p63 and intense nuclear expression (20%) of Ki-67 (Fig. 3). There was a lack of expression of CK5/6, CD10, acute myeloid leukemia (AML), and BCL2. The tumor was consistently unreactive to estrogen receptor (ER) and progesterone receptor (PR) and did not express human epidermal growth factor receptor 2 (HER2).

The final diagnosis was SCC (variant of metaplastic carcinoma according to the WHO 2012 classification). For the clinical staging, a thoracoabdominal pelvic computed tomographic scan and bone scintigraphy were performed, which did not show distant metastases. The tumor was classified pT2N0M0 according to the classification system of the American Joint Committee on Cancer, seventh edition [4]. Radiation therapy with a total dose of 50 Gy was administered. The patient developed grade 3 radiation dermatitis. No endocrine treatment or chemotherapy was administered. Surveillance consisted of a clinical assessment every 3 months and a mammogram every year. No recurrence was reported after 8 months of follow-up.

## Discussion

Spindle cell lesions of the breast refer to a very diverse group with biological heterogeneity and may be benign or malignant [4]. They are relatively rare and cause diagnostic difficulty. Among these lesions, SCC is a special subtype of breast carcinoma. It represents a variant of metaplastic carcinoma according to the fourth edition of the WHO classification of tumors of the breast, published in 2012 [1]. The origin of SCC has long been a subject of debate, and several terms have been used in the past, such as carcinosarcoma, metaplastic carcinoma, and metaplasic carcinoma [2]. SCC of the breast remains a rare disease, and only a few cases have been reported in the literature [3].

SCC occurs in postmenopausal women, and the average age of presentation is 55 years. Clinically, it presents as a palpable and large mass (larger than T3 in 50% of cases). Khan *et al*. [2] demonstrated that the imaging features of various subtypes of metaplastic carcinoma have certain characteristics. Usually, SCC presents as an oval-shaped mass with circumscribed margins and a slightly high density classified as BI-RADS 4 or BI-RADS 5. Microcalcifications visualized by mammography are uncommon [2]. In our patient, the lesion measured 3 cm and was a nodule with irregular contours associated with microcalcifications. It was classified as BI-RADS 4 on the basis of mammography.

The prognosis of SCC is unclear, and results vary considerably from one study to another. The reported 5-year survival rate ranges from 28% to 68% [5, 6]. The tumor diameter and grade seem to be the most important prognostic factors. SCC is less likely than common subtypes of ductal or lobular breast carcinomas to have lymph nodes (estimated at around 6%) and distant metastases [7]. In our patient, no lymph node involvement or distant metastases were found.

Macroscopically, these tumors are generally well defined with focal marginal irregularity. There may be a necrotic focus and hemorrhage on the firm, rubbery cut surface in larger tumors, and sometimes nodular areas of hyalinization may be present, even in smaller tumors [4]. The histology of SCC is rather peculiar and characterized by the presence of a pure or dominant population of a spindle cell island with some other component of squamous cells, lobular carcinoma, and ductal carcinoma. Microscopic examination reveals an infiltrating proliferation of spindle cells lacking significant atypical or mitotic activity, which typically exhibits epithelial differentiation [8, 9].

Immunohistochemistry is the key examination that allows more accurate diagnosis. Specific markers have high sensitivity and specificity for spindle cells and are useful for the diagnosis of SCC. Findings of focal positivity for cytokeratin (AE1/AE3, CK5/6, CK7, and CK14) and the presence of S100 protein are in favor of this lesion. There may be a positive reaction for muscle markers such as calponin (86%) and smooth muscle actin (36%) [10, 11]. p63 is a sensitive and relatively specific marker for epithelial cells [12, 13]. In our patient, the tumor cells were immunoreactive for cytokeratin (AE1/AE3) and p63.

SCC is typically a triple-negative tumor, which limits the therapeutic options. In Moten *et al*.’s study, in which they reviewed all cases of cancer published from 1992 to 2011 (286 cases), only 15% were ER-positive [14]. In addition, researchers in a recent study reported positive status of 5.2%, 5.2%, and 10.5% for ER, PR, and HER2, respectively [15].

Histopathologically, it is difficult to make a diagnosis of SCC, and SCC must be considered in the differential diagnosis of other spindle cell lesions (benign and malignant entities), such as fibromatosis, primary low-grade sarcoma, inflammatory myofibroblastic tumor, and malignant phyllodes tumors (MPTs) [8, 15]. However, the development of immunohistochemistry has led to better knowledge of this histological type, despite its rarity. The diagnosis is usually established using antibodies to keratin of variable molecular weight.

Fibromatosis is a benign spindle cell lesion with minimal pleomorphism and either absent or minimal mitosis. It often expresses actin and occasionally desmin and S100, whereas in contrast to SCC, CKs are not expressed [16]. Primary pure spindle cell sarcomatoid carcinoma is a rare disease, and the establishment of this diagnosis is difficult in adults. IHC examination shows a lack of epithelial components in most pure spindle cell sarcomas of the breast [17]. MPTs are fibroepithelial tumors characterized by the phyllodes pattern. Generally, the diagnosis of MPT does not require the use of immunohistochemistry [15, 18].

SCC exhibits a propensity for local recurrence. The prognostic factors for SCC are not well known. Researchers in a multinational study of prognostic factors in metaplastic carcinoma of the breast found that this breast cancer entity is a heterogeneous disease encompassing biologically different tumor classes with variable outcomes. SCC was associated with worse prognosis than matrix-producing and squamous carcinomas [19]. Another significant prognostic factor in this study was tumor size. In a literature review in which the authors reported long-term outcomes of SCC of the breast, it was found that tumor size (greater than 2 cm) appeared to be a significant risk factor [20]. Recently, it has been reported that p53 and p63 accumulated mutations are potential markers for a high risk of malignancy and prognosis in SCC [21].

Owing to the rarity of SCC, very little is known about its biological behavior and systemic treatment. The management of SCC is problematic, and no standard treatment is established. A wide excision with adequate margins is an appropriate treatment in selected patients (tumor diameter smaller than 2 cm). Mastectomy could be considered in patients with significant tumor size [22]. However, the results of studies are contradictory. On one hand, in a comprehensive analysis of SCC of the breast, researchers found that the proportion of patients who underwent partial mastectomy increased over time and that the 10-year survival rates were 82.5% for patients who underwent partial mastectomy and 61.5% for those who underwent complete mastectomy. However, because patients with late-stage disease have a poor prognosis compared with those with early-stage disease, a subgroup analysis was done. The conclusion was that there was no significant difference in survival between patients who underwent partial mastectomy from that of patients who underwent total mastectomy [14]. On the other hand, Song *et al*. showed that local tumor recurrence was more frequent with simple mastectomy than with radical mastectomy [23]. The prognosis and survival rate were better with radical mastectomy and modified radical mastectomy. In our patient, a radical mastectomy was performed. In general, there is no lymph node invasion in this variant of metaplastic carcinoma; nevertheless, sentinel lymph node biopsy is used to determine staging.

The use of radiotherapy in SCC remains unclear. The data reported in the literature are contradictory. Some authors suggest that radiation can be performed to minimize local recurrence mainly in patients with high risk. By contrast, Moten *et al*. found that among patients with late-stage disease, those who underwent complete mastectomy with adjuvant radiation had worse survival rates than those who underwent complete mastectomy alone, but not to a statistically significant degree [14]. In addition, Tseng *et al*. suggested that adjuvant radiation improved survival for all patients with metastatic breast cancer, regardless of type of surgery [24]. Our patient received radiotherapy. She did not undergo any other further treatment.

Hormone therapy (tamoxifen or aromatase inhibitor) and targeted therapy (anti-HER2) have significantly improved progression-free survival and overall survival in patients with breast cancer. However, SCC is generally triple-negative, and the effectiveness of hormone therapy or targeted therapy is unknown [25].

The use of chemotherapy and which agent is used are not well defined. Currently, there is no indication for adjuvant chemotherapy. This remains the case with local recurrence and distant metastasis [26]. Authors reporting some rare published cases have evaluated chemotherapy combinations. In neoadjuvant situations, chemotherapy did not show effectiveness in reducing tumor size and preventing progression of disease [27, 28]. The protocols usually used for breast cancer have not shown efficacy (anthracycline, vinorelbine, cyclophosphamide, and taxanes). In addition, in metastatic cases, a low response rate with chemotherapy has been observed [29]. Because the epidermal growth factor receptor is expressed in > 90% of SCC cases, it has been supposed that tyrosine kinase inhibitors can be effective for treatment of this subtype [30]. In this context, Zhou *et al*. tested apatinib, a tyrosine kinase inhibitor targeting vascular endothelial growth factor receptor 2, in a patient with advanced SCC of the breast. The results showed a nearly complete response as well as controllable and tolerable side effects [31]. However, additional clinical trials are needed. A multidisciplinary treatment approach is recommended for the management of metaplastic SCC, taking into account all the particularities of this rare type.

## Conclusions

We describe a case of a patient with SCC of the breast, which is an extremely rare tumor entity. The diagnosis of SCC is difficult and requires a rigorous histopathological approach and IHC examination must be done to confirm the diagnosis. The long-term prognosis of SCC remains unknown because of the paucity of reported cases with long-term follow-up. However, SCC is characterized by a high risk of local recurrence, hence the importance of adequate care. Currently, the role of chemotherapy is not clear, and it is necessary to study other cases before establishing a consensus for this type of tumor. We will continue to follow our patient to obtain long-term survival data.

## Abbreviations

BI-RADS: Breast Imaging Reporting and Data System
ER: Estrogen receptor
HER2: Human epidermal growth factor receptor 2
IHC: Immunohistochemical
MPT: Malignant phyllodes tumor
PR: Progesterone receptor
SCC: Spindle cell carcinoma
WHO: World Health Organization
